# Measurement and application of patient similarity in personalized predictive modeling based on electronic medical records

**DOI:** 10.1186/s12938-019-0718-2

**Published:** 2019-10-11

**Authors:** Ni Wang, Yanqun Huang, Honglei Liu, Xiaolu Fei, Lan Wei, Xiangkun Zhao, Hui Chen

**Affiliations:** 1 School of Biomedical Engineering, Capital Medical University, No. 10, Xitoutiao, YouAnMen, Fengtai District, Beijing, 100069 China; 2 Beijing Key Laboratory of Fundamental Research on Biomechanics in Clinical Application, Capital Medical University, No. 10, Xitoutiao, YouAnMen, Fengtai District, Beijing, 100069 China; 3 Information Center, Xuanwu Hospital, Capital Medical University, No. 45 Changchun Street, Xicheng District, Beijing, 100053 China

**Keywords:** Patient similarity, Electronic medical records, Personalized prediction, Model performance, Diabetes mellitus

## Abstract

**Background:**

Conventional risk prediction techniques may not be the most suitable approach for personalized prediction for individual patients. Therefore, individualized predictive modeling based on similar patients has emerged. This study aimed to propose a comprehensive measurement of patient similarity using real-world electronic medical records data, and evaluate the effectiveness of the individualized prediction of a patient’s diabetes status based on the patient similarity.

**Results:**

When using no more than 30% of the whole training sample, the personalized predictive models outperformed corresponding traditional models built on randomly selected training samples of the same size as the personalized models (*P* < 0.001 for all). With only the top 1000 (10%), 700 (7%) and 1400 (14%) similar samples, personalized random forest, *k*-nearest neighbor and logistic regression models reached the globally optimal performance with the area under the receiver-operating characteristic (ROC) curve of 0.90, 0.82 and 0.89, respectively.

**Conclusions:**

The proposed patient similarity measurement was effective when developing personalized predictive models. The successful application of patient similarity in predicting a patient’s diabetes status provided useful references for diagnostic decision-making support by investigating the evidence on similar patients.

## Background

In personalized medicine, clinicians and health policy makers must choose the most appropriate clinical trial and make predictions for the right patient during decision-making [1, 2]. This approach is used to individualize medical practice.

At present, clinicians can predict diseases by many methods like diagnostic imaging technique [3–7] but with fewer predictive models. In recent years, predictive modeling has been successfully applied in the medical scenarios, including the identification of risk factors [8, 9] and early detection of disease onset [10, 11]. In addition, advances have been made in using predictive modeling to predict patient outcomes [2]. The traditional predictive modeling approach involves building a global predictive model using all available training data. However, this may not be the most suitable approach for personalized prediction for individual patients. Furthermore, generally there are varieties of noisy data in electronic medical records (EMR) data, which were primarily designed for administration and improving healthcare efficiency, and many studies have found secondary use such as patient trajectory modeling, disease inference and clinical decision support system [12]. It is recommended to de-noise data before building a global predictive model, which will be time consuming and challenging to represent and model. In this context, individualized predictive modeling based on patient similarity emerged and was shown to be adjustable for individual patients. Employing patient similarity helps to identify a precision cohort for an index patient, which will then be used to train a personalized model [2]. Accordingly, when building a predictive model for an index patient, training samples are determined as “patients like me,” instead of using all available training samples in a conventional way. “Patients like me” are selected from the training sample set on the basis of similarity between the index patient and each training sample. Of note, based on patient similarity, patients with noisy data are less likely to be selected as similar patients of an index patient for the reason of the less similarity between them. Patient similarity is usually measured by considering information on demographics, disease history, comorbidities, laboratory tests, hospitalizations, treatment, and pharmacotherapy. Such data are easily extracted from the EMR for tens of millions of patients [13].

In this study, we defined a patient as a vector in a d-dimensional feature space. Then, a multi-dimensional approach to estimate patient similarity was proposed. To demonstrate the effectiveness of the proposed similarity measure, the most similar patients were retrieved to build personalized models to predict the diabetes status of a given patient.

### Related work

To assist physicians with the selection of the most appropriate recommendations and the prediction of a given patient, several methodologies have been applied in personalized medicine such as clustering, principle component analysis and patient similarity computation.

Clustering is the most popular method used in personalized medicine. This aims to create groups of patients with similar disease evolution [14], with the prediction for a new patient identified with the label of their most similar cluster. To determine the subtype for a breast cancer patient and provide the most effective treatment, Wang et al. [15] defined a novel consensus clustering method to automatically cluster numerical and categorical data using Euclidean distance and categorical distance, respectively. The proposed method demonstrated great superiority and robustness in clustering and differentiating patient outcomes. Li et al. [16] presented an unsupervised clustering framework based on topological analysis to identify type 2 diabetes subgroups. The topology-based patient–patient network could be used for identifying three distinct subgroups of type 2 diabetes successfully. Panahiazar et al. [17] designed two different approaches for medication recommendation for a heart-failure patient, using both unsupervised clustering (hierarchical clustering and K-means clustering) and supervised clustering (using the medication plan as class variable). Their results showed that supervised clustering outperformed the unsupervised clustering.

Another frequently used technique for predicting patient outcomes is based on the patient similarity. Patient similarity evaluation was investigated as a tool to enable precision medicine [14], and was identified as a fundamental problem in many data mining algorithms and practical information process systems [18]. Most commonly, through exhaustive comparisons between a given patient and a cohort of existing patients, an assessment specific to the given patient can help in identifying his similar patients. Lee et al. [19] used a cosine-based patient similarity metric to identify patients who agreed the most with each patient. The result suggested that using fewer but more similar data could get higher predictive performance than using overall available data. David et al. [20] proposed an algorithm for the anomaly detection and characterization on the basis of the Euclidean distance between the medical laboratory data. With the selected neighbors around him, the index patient could be segmented into one of the seven disease groups with a higher accuracy. For early screening and assessment of suicidal risks, researchers used the sum of absolute distances for each predictor to retrieve a cohort of similar patients and determined the most potential risk level for a new patient [21]. Among these studies, one of them [19] compared the performance of the patient similarity-based personalized predictive models with the whole population-based global predictive models. The results demonstrated that personalized predictive models showed a higher performance.

Many previous studies usually calculated the patient similarity using single similarity measures (e.g., Euclidean distance, cosine distance, and Mahalanobis distance), and most of them did not take the importance of patient features into consideration while calculating the similarity. In this study, we aimed to investigate in depth the patient similarity in the following two aspects. One is using different similarity metrics for different types of feature data. The other is assigning different weights (importance) to patient features when integrating feature similarities into a patient similarity.

## Results

### Overview of patient similarity

**Fig. 1:**
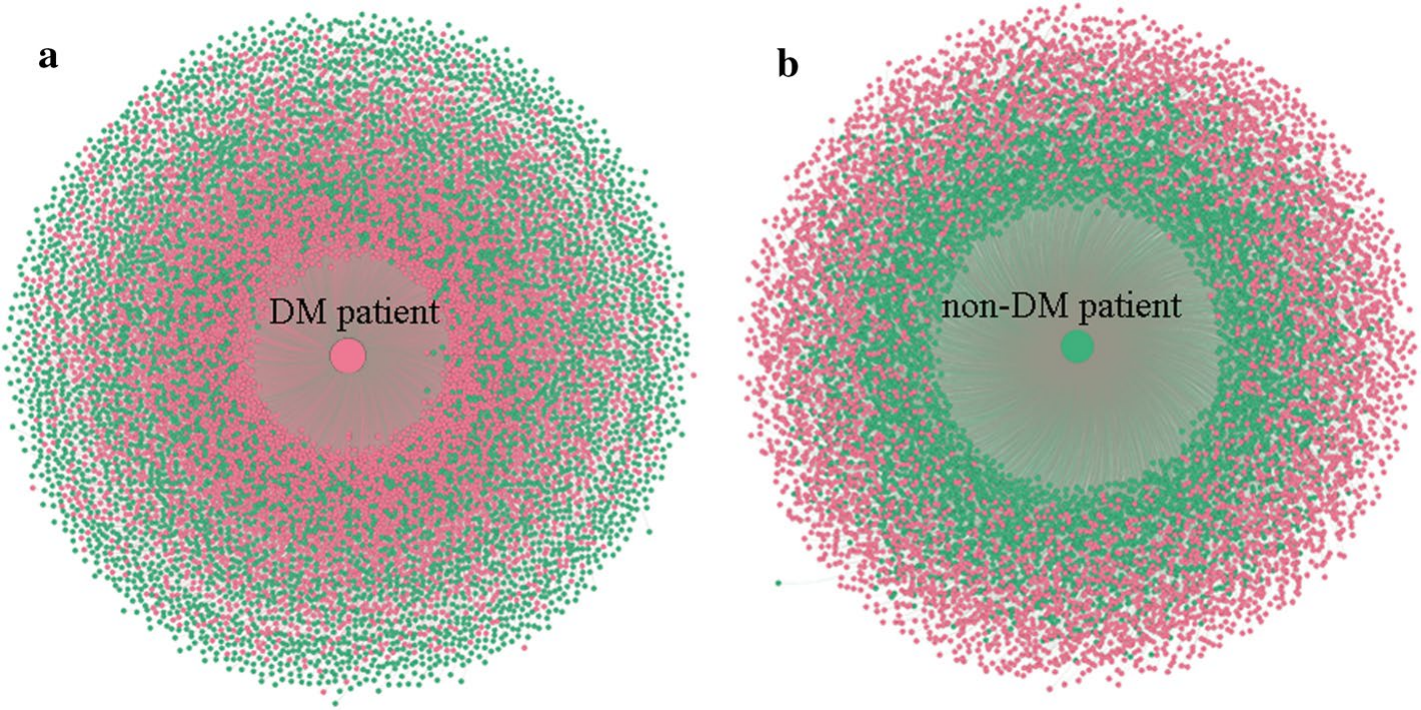
Visualization of patient similarity when the feature similarity for disease diagnosis was calculated using International Classification of Diseases, tenth revision (ICD-10) disease codes. The central big dots represent two index patients from the test sample set [red for a patient with diabetes mellitus (DM) and green for a patient without DM]. The surrounding dots represent all patients with DM (red) and without DM (green) from the training sample set, where the distance to the central dot corresponds to the similarity. The closer the surrounding dots are to the central dot, the more similar are the two patients

To validate the predictive performance of the patient similarity-based models, we calculated all possible similarities between each pair of patients (one selected from the test set and the other from the training set). In the distribution scatter plot (Fig. 1) of similarity measurements for a patient with diabetes mellitus (DM), other patients with DM were more likely to be closer to the index patient than patients without DM (Fig. 1a). There was a similar trend in the distribution scatter plot for a patient without DM (Fig. 1b).

**Fig. 2:**
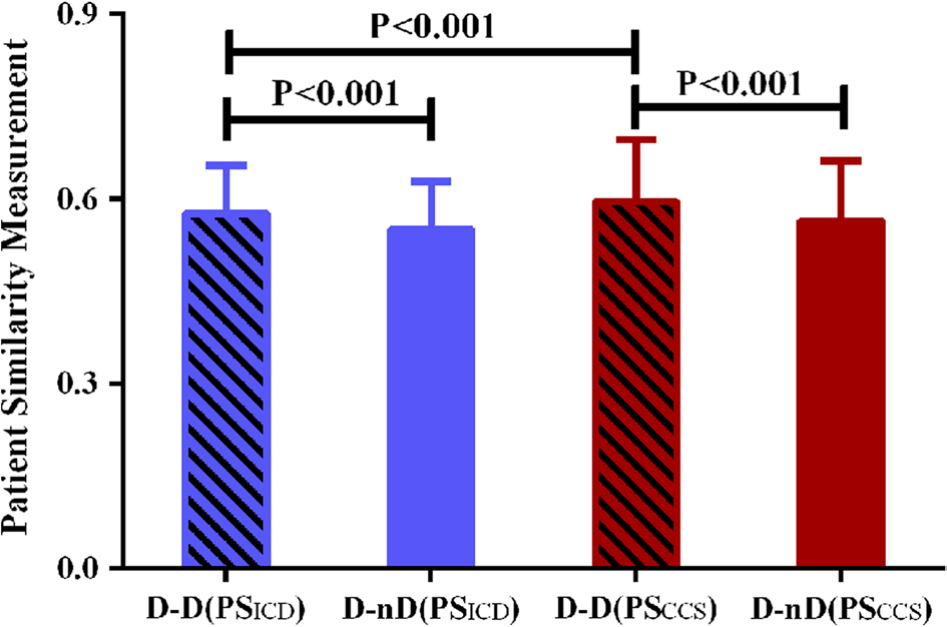
Patient similarity among patients with and without diabetes. D-D(ICD-based similarity) and D-D(CCS-based similarity) represent similarities between pairs of patients with diabetes mellitus (DM) based on ICD-10 and CCS disease codes, respectively. Error bars represent standard deviation. D-nD(ICD-based similarity) and D-nD(CCS-based similarity) represent similarities between patient pairs that included at least one patient without DM based on ICD-10 and CCS disease codes, respectively

On average, similarities between pairs of patients with DM [0.576 ± 0.078 calculated by Eq. 7 and 0.596 ± 0.100 calculated by Eq. 8, respectively] were both statistically greater than those between patient pairs that included at least one patient without DM (0.550 ± 0.078 and 0.565 ± 0.097, respectively; *t* test, *P* values < 0.001 for both). International Classification of Diseases, tenth revision (ICD-10) codes-based similarities among patients with DM were less than Clinical Classification Software (CCS) codes-based similarities (*t*-test, *P* < 0.001; Fig. 2).

### Evaluation of predictive performance

**Fig. 3:**
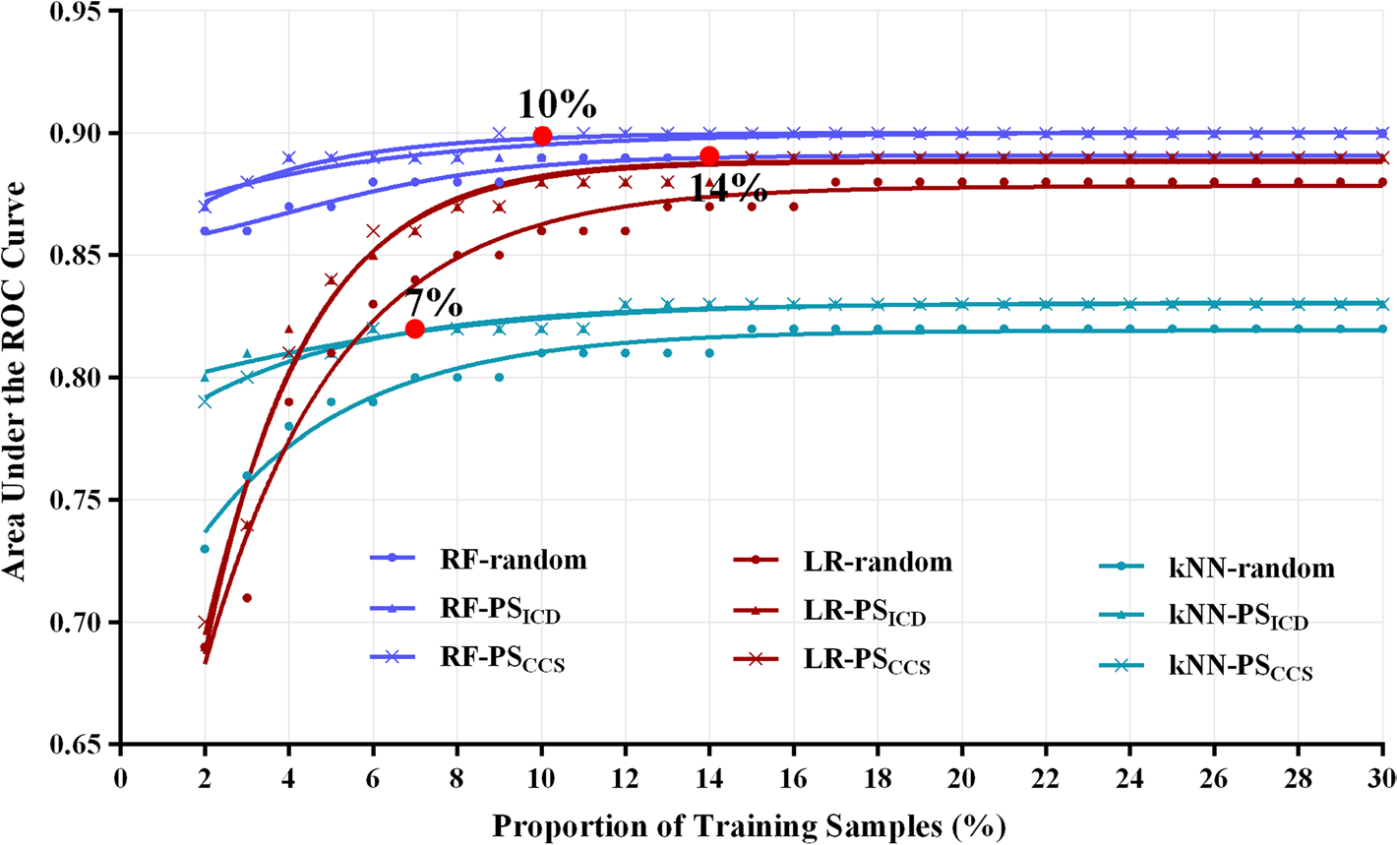
Predictive performance of random forest (RF), logistic regression (LR), and *k*-nearest neighbor (*k*NN) models. For simplicity, only performances of the models built on 2% (200 samples) to 30% (3000 samples) of the 10,000 training sample candidates are displayed in the figure. Blue, cyan, and dark red lines represent RF, *k*NN, and LR models, respectively. Lines with dot, triangle, and cross markers represent models built on the randomly selected samples and the most similar samples based on patient similarity when the similarity of disease diagnoses feature was calculated using ICD-10 and CCS codes

When no more than 30% of the whole training sample (i.e., 3000 samples) were used to build the models, all three personalized predictive models outperformed the corresponding traditional models, which were built on randomly selected training samples of the same size as the personalized models (Mann–Whitney *U* test adjusted by Bonferroni, *P* values < 0.001 for all). As the number of training samples increased, the personalized and traditional predictive models showed almost the same globally optimal performance. However, only the top 1000 (10%), 700 (7%), and 1400 (14%) similar samples were used for building the personalized random forest (RF), k-nearest neighbor (*k*NN), and logistic regression (LR) models, respectively, while 3600 (36%), 1400 (14%), and 3700 (37%) random selected samples were used for the corresponding traditional models (Fig. 3). This suggested that the personalized models reached the optimal performance using fewer, but more similar training samples.

When the top 1000 (10%), 700 (7%), and 1400 (14%) similar samples selected according to the CCS-based similarity were used, the personalized RF, *k*NN, and LR models showed a clear increasing trend from the initial area under the receiver-operating characteristic (ROC) curve of 0.87, 0.79, and 0.70 to the saturated area under the ROC curve (AUC) of 0.90, 0.82, and 0.89, respectively. When the *k*NN model was built using up to the top 4% of similar samples, it outperformed the LR model. This suggested that more appropriate data were needed for the LR model parameters to be properly trained. Similar results were found when patient similarities were based on ICD-based similarity. When RF, *k*NN, and LR models were built on the top 12%, 7%, and 15% of similar samples, respectively, they showed the globally optimal performance. The RF model showed significantly higher performance than the LR and *k*NN models (Mann–Whitney *U* test adjusted by Bonferroni, *P* values < 0.001 for all), partially because of its built-in feature selection property.

Further comparisons of predictive performance of the personalized models built on ICD-10- and CCS-based similar patients showed that there were no significant differences for RF, *k*NN, and LR models (Mann–Whitney *U* test adjusted by Bonferroni, *P* = 0.491, 0.988 and 0.635, separately).

### Interpretation of predictive models

**Fig. 4:**
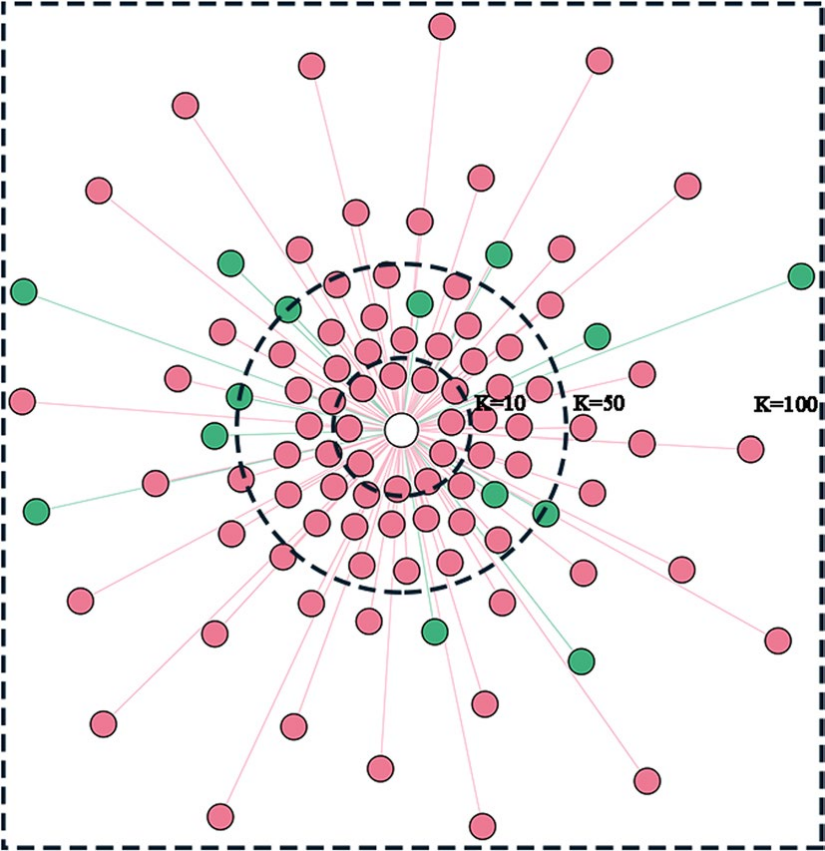
The visualized classification process of the *k*-nearest neighbor (*k*NN) model for a randomly selected index patient. The *k* represents the number of nearest neighbors. The central circle represents an index patient from the test sample set. The surrounding dots represent *k*-nearest neighbors with DM (red) and without DM (green) from the training sample set, where the distance to the central circle corresponds to the Euclidean distance

The visualized classification process of the *k*NN model for a randomly selected index patient (a true DM patient, the central circle) is shown in Fig. 4. No matter what the parameter *k* was set, the index patient was always predicted to be a DM patient. For example, there were 100% (10/10), 94% (47/50) and 86% (86/100) patients with DM (red dots) among the index patient’s 10, 50 and 100 nearest neighbors (i.e. *k* = 10, 50, and 100), respectively.

**Fig. 5:**
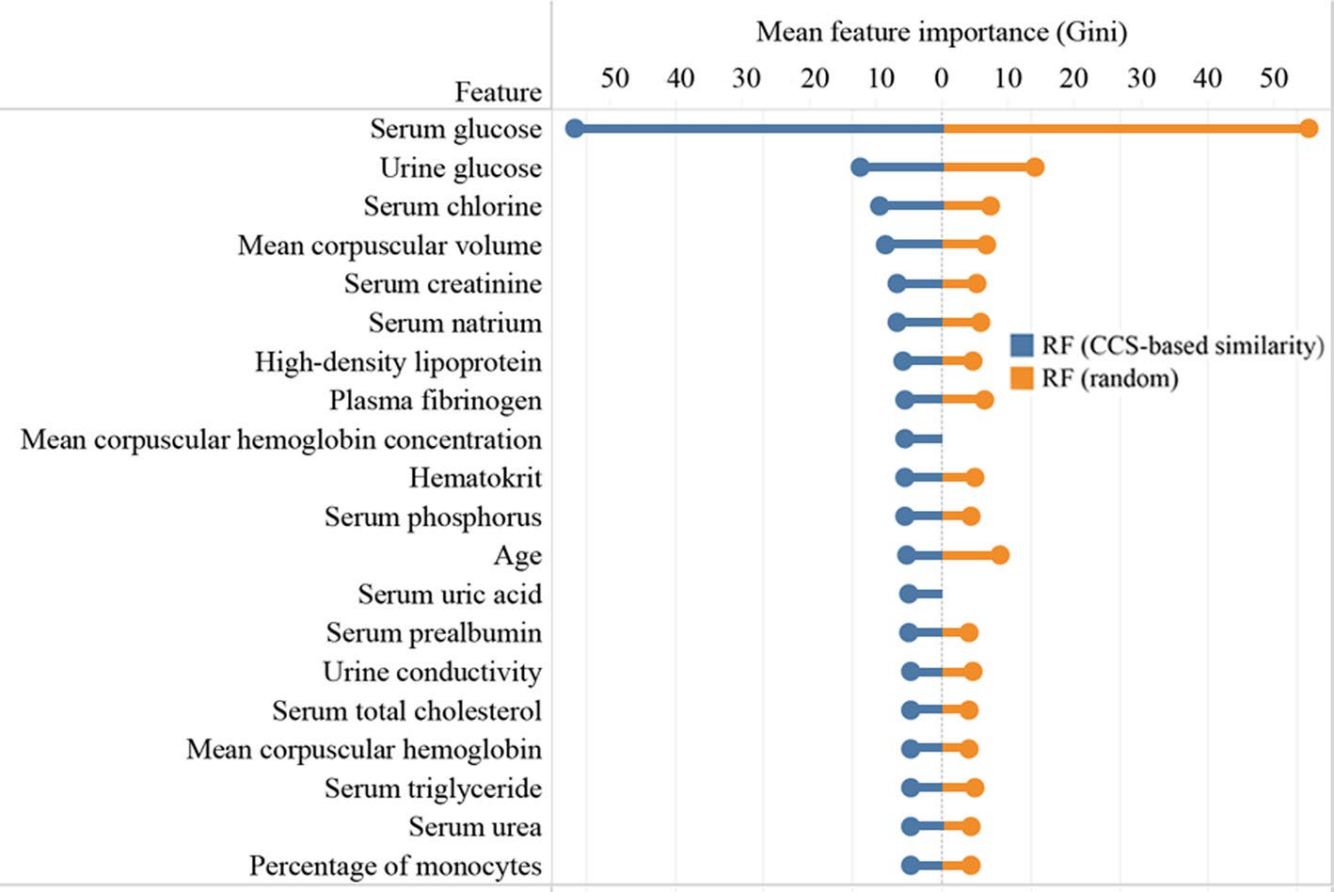
The plot showing the top 20 important features for diabetes identified by the random forest (RF) model according to Gini coefficients. Dark blue and orange columns represent RF model built on similar samples selected according to the CCS-based similarity and randomly selected samples, respectively

Since the RF model provided the highest predictive performance in this study, feature importance obtained from the RF models was presented to help understanding the model (Fig. 5). The top 20 important features for diabetes prediction included one demographic characteristic (i.e., age) and several laboratory tests (such as serum glucose, urine glucose, and serum chlorine). Features’ importance varied with the training samples (similar samples or randomly selected samples) on which RF models were built.

## Discussion

Prediction of risk for specific diseases is important in a variety of applications, including health insurance, tailored health communication, and public health [22]. In this paper, we proposed a method for predicting risk for a potential disease using a large clinical dataset collected from an EMR system. In the proposed method, classification algorithms (*k*NN, LR, and RF) were built to predict a patient’s diabetes status based on patient similarities assessed using a multi-dimensional approach covering demographics, disease diagnoses, and laboratory tests. The investigation pipeline can easily be extended to the study of other complex and multifactorial diseases.

Because patients’ disease diagnoses were an important part of EMR data and a key factor for disease prediction, we investigated two similarity measurements for disease diagnoses. One was calculated using a hierarchical similarity measure with ICD-10 disease codes, and the other using simple cosine similarity with CCS disease codes. Although the hierarchical similarity measure has been argued to be a more direct mapping of hierarchical information to distances [23], we found that predictive models built on the most similar samples selected according to patient similarity based on hierarchical similarity did not show higher performance than those based on cosine disease similarity. This suggests that narrowing ICD-10 diagnosis codes into CCS codes may be useful for presenting disease data at a descriptive statistical categorical level [16]. Therefore, feature similarity for disease diagnoses based on CCS codes and cosine similarity was more effective and efficient than that based on ICD-10 codes and hierarchical similarity in this study.

A previous study suggested that in personalized medicine, using patient similarity in data-driven analysis of patient cohorts will significantly assist physicians to make informed decisions and choose the most appropriate clinical trial [24]. In this study, three different predictive models using similar cohorts showed a consistently higher performance, especially in that they used fewer training samples than those built on randomly selected samples. This finding coincided with the conclusion that similarity-based selection was better than random selection [8]. In particular, the personalized LR model showed the largest performance increase. This demonstrated that patient similarity has potential to improve the predictive performance of machine learning models.

Furthermore, predictive performance for both the personalized and traditional models reached a saturated level when increasing numbers of training samples were involved in the modeling, where the personalized models reached earlier. This finding was consistent with the conclusion of two previous studies that little was gained from using more dissimilar patients when building models [8, 25]. Generally, there are varieties of noisy data (errors) in EMR, where noisy data referred to the irrelevant and dissimilar data for a patient with the specific disease. When building personalized models, the most similar samples measured by the proposed patient similarity were used as the training samples, which could be considered as “the patients like me”. Under this situation, noisy data which may disturb the prediction were less likely to be selected as training samples due to the less similarity; thus, patient similarity measurement proposed herein could be harnessed as a de-noising method. This improved the predictive performance and the overall robustness of aforementioned models to some degree. Using fewer but more similar samples, personalized predictive models may perform as well as traditional predictive models built on the entire training samples. For the personalized models, as the training sample size increased, more and more samples with less similarity were added into the training set, making the overlap of training set for the personalized models and traditional models enlarged. When the training sample size increased to the whole available training samples, no difference would exist in the similarity-based selection and random selection of training samples. The personalized models, thus, degenerated into the traditional ones, both showing the same predictive performance, the global performance.

Diabetes prediction is a challenging task for its multifactorial characteristics and various manifestations. Park et al. [25] applied their new knowledge discovery techniques to improve the performance of diabetes prediction, obtaining an average accuracy of 0.76. In another study [8] of diabetes prediction, the best performance (AUC, 0.62) of the personalized models was obtained when the predictive model was built on 2000 similar patients. In our study, based on the proposed similarity measurement, predictive performances for diabetes improved a lot with the highest AUC of 0.90.

There are some limitations to our research. First, when constructing study cohort, no exclusion criterion specific to the predictive task was employed. Second, the patient similarity was calculated directly, without making the full use of the information provided by the large amount of sample patients. Last, the performance of the proposed patient similarity measure was only evaluated for disease prediction. In the further work, we will improve the algorithm for the similarity measurement, including learning the patient similarity automatically, and the patient similarity will be used in other application scenarios, such as patient stratification for disease sub-typing.

## Conclusions

In this study, we proposed a comprehensive measurement of patient similarity using real-world EMR data, and evaluated the effectiveness of the individualized prediction of a patient’s diabetes status based on the patient similarity. The proposed similarity measure was designed to reflect the data type and clinical meaning of each patient feature. Moreover, predictive models built on similar cohorts had a consistently higher performance than those built on randomly selected samples. They also performed as well as models built on entire training samples. This makes it possible for further large-scale and high-dimensional predictive applications at relatively lower time and space costs and higher performance. The successful application of patient similarity in predicting a patient’s diabetes status provided useful references for diagnostic decision-making support by investigating the evidence on similar patients.

## Methods

**Fig. 6:**
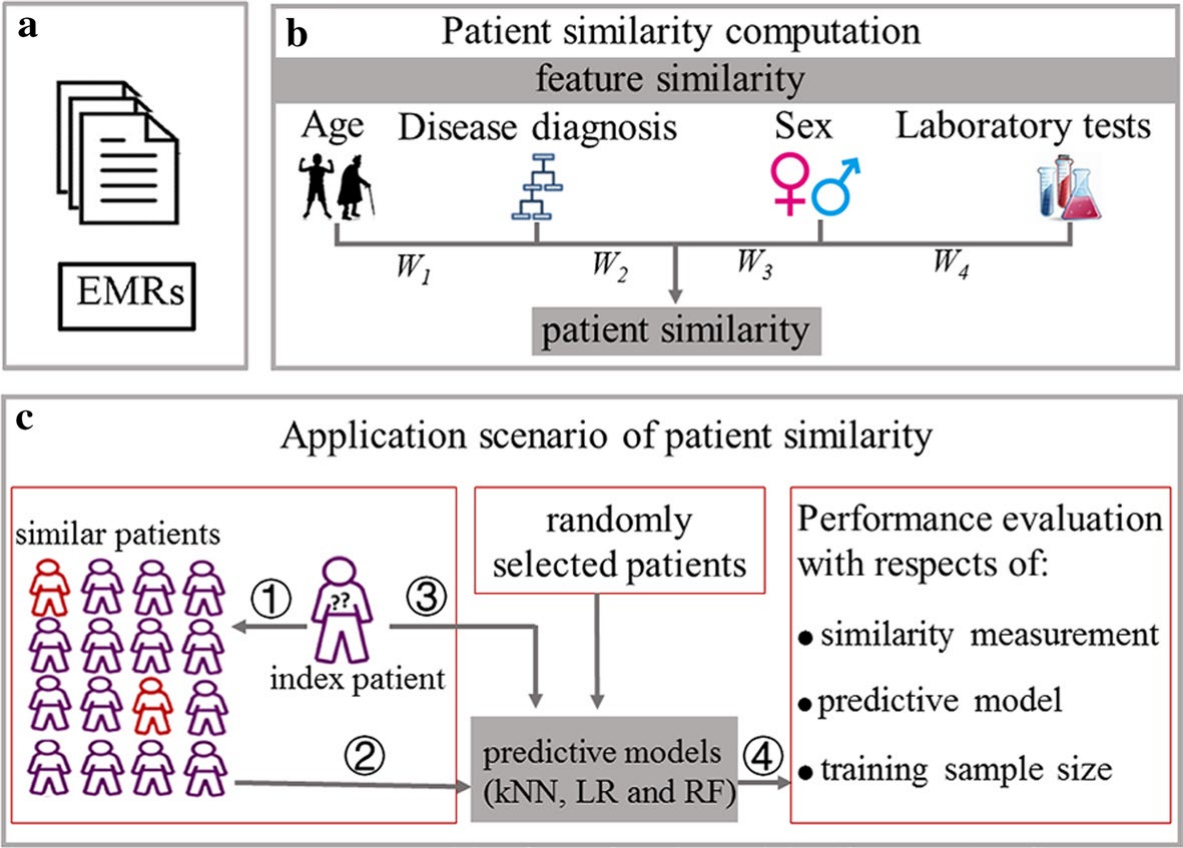
Main steps of the workflow. **a** Retrieving analyzed data from EMRs data. **b** Calculation of four types of feature similarities and patient similarity. **c** Application of patient similarity into personalized predictive model for future diabetes status prediction. *kNN k*-nearest neighbor, *LR* logistic regression, *RF* random forest, *EMRs* electrical medical records

In this study, patient similarity was estimated using four types of patient information or features: age, sex, multiple laboratory test items, and multiple disease diagnoses. Similarities were first calculated at the feature level, and then combined into a single similarity measure at the patient level. The main steps of the workflow are shown in Fig. 6.

### Similarity calculation

#### Feature similarity for age

Let Age_*i*_ and Age_*j*_ denote the age of patients *i* and *j*, respectively. The feature similarity for age (FS_*A*_) was defined as the ratio of the smaller age value to the larger one:

1 $${\text{FS}}_{A} \left( {i, j} \right) = \frac{{\hbox{min} \left( {{\text{Age}}_{i} ,{\text{Age}}_{j} } \right)}}{{\hbox{max} \left( {{\text{Age}}_{i} ,{\text{Age}}_{j} } \right)}}.$$

#### Feature similarity for sex

The feature similarity for sex (FS_*S*_) between patients *i* and *j* was defined as 1 if the two patients had the same sex and 0 otherwise.

2 $${\text{FS}}_{S} \left( {i,j} \right) = \left\{ {\begin{array}{*{20}c} {1,} & {{\text{if}}\;{\text{patients}}\;i\;{\text{and}}\;j\;{\text{had}}\;{\text{the}}\;{\text{same}}\;{\text{sex}}} \\ {0,} & {\text{otherwise}} \\ \end{array} } \right..$$

#### Feature similarity for laboratory test

All *m* laboratory test items had continuous values in the EMR in this study. They were first normalized to *L*_*xy*_ ~ *N* (0,1) for the further calculation, where *L*_*xy*_ represents the normalized lab test *y* for patient *x*. The feature similarity for lab test (FS_*L*_) was defined as 1 minus the normalized Euclidean distance (by min–max normalization), as shown in Eqs. (3) to (5).

3 $$d_{\text{lab}} \left( {i, j} \right) = \sqrt {\left( {L_{i1} - L_{j1} } \right)^{2} + \left( {L_{i2} - L_{j2} } \right)^{2} + \cdots + \left( {L_{im} - L_{jm} } \right)^{2} }$$

4 $$d^{\prime} = \frac{{d_{\text{lab}} \left( {i, j} \right) - \hbox{min} \left( {d_{\text{lab}} } \right)}}{{\hbox{max} \left( {d_{\text{lab}} } \right) - \hbox{min} \left( {d_{\text{lab}} } \right)}}$$

5 $${\text{FS}}_{L} \left( {i, j} \right) = 1 - d^{\prime}.$$

#### Feature similarity for disease diagnoses

Disease diagnoses were initially identified using ICD-10 codes [26]. In the ICD-10 code scheme, each code begins with a letter (A–Z for 22 chapters) followed by five digits, arranged in a tree-like hierarchical manner (Additional file 1: Figure S1). The letter and first three digits are usually used for statistical purposes [16]; they were, therefore, used to calculate feature similarity for disease diagnosis in this study. As an alternative to the ICD-10 code scheme, the CCS code scheme [27] collapsed ICD-10 codes into 259 diagnosis codes (numbered 1–259) with better generalization and clinical meaningfulness [16]. For example, DM was designated as ICD-10 codes E10.x–E14.x; corresponding CCS codes were 49 (DM without complications) and 50 (DM with complications).

We proposed two methods of measuring disease diagnosis similarity based on the two code schemes with totally different structures.

#### Feature similarity for disease diagnoses based on the ICD-10 code scheme

Considering the path distance between concepts (nodes) in the ICD-10 hierarchy system, the similarity *S*(*x*, *y*) between two single codes *x* and *y* was calculated using the level of their nearest common ancestor (NCA) over the level of themselves in the hierarchy system, as shown in Eq. (6) [28].

6 $$S\left( {x,y} \right) = \frac{{{\text{NCA}}\left( {x,y} \right)}}{{\# {\text{levels}}}},$$

where #levels is the number of levels in the ICD-10 hierarchy system. For example, the level of ICD-10 codes E10.9 and E11.9 was 4, and the level of their NCA (i.e., E1) was 2; therefore, the similarity of the two diagnoses was calculated as 2/4 = 0.5.

Two patients were considered similar if their sets of diagnoses overlapped, and more similar if they showed a greater degree of overlap. For two ICD-10 code sets, *X* = {*x*_1_, *x*_2_, … *x*_*l*_} for patient *i* and *Y* = {*y*_1_, *y*_2_, … *y*_*n*_} for patient *j*, only the elements in the intersection of the two sets were considered when calculating similarity. The feature similarity for disease diagnosis represented by ICD-10 codes (FS_D1_) was defined in Eq. (7) [23]:

7 $${\text{FS}}_{{{\text{D}}1}} \left( {i,j} \right) = 1 - \frac{1}{{\left| {X \cup Y} \right|}}\left( {\sum\nolimits_{{x_{l} \in X\backslash Y}} {\frac{1}{\left| Y \right|}\sum\nolimits_{{y_{n} \in Y}} {d\left( {x_{l} ,y_{n} } \right) + \sum\nolimits_{{y_{n} \in Y\backslash X}} {\frac{1}{\left| X \right|}\sum\nolimits_{{x_{l} \in X}} {d(y_{n} ,x_{l} } } } } } \right),$$

where $d\left( {x,y} \right) = d\left( {y,x} \right) = 1 - S\left( {x,y} \right)$ in Eq. (6).

#### Feature similarity for disease diagnoses based on the CCS code scheme

For patient *X*, disease diagnoses were represented by a 259-dimensional 0–1 vector *X* = {*x*_1_, *x*_2_, … *x*_259_}, where *x*_*n*_ = 1 if the patient had the disease represented by the CCS code *k*, and 0 otherwise. Feature similarity for disease diagnosis represented by CCS codes (FS_D2_) was defined as the cosine similarity between CCS code vectors *X* for patient *i* and *Y* for patient *j* (Eq. 8).

8 $${\text{FS}}_{{{\text{D}}2}} \left( {i, j} \right) = \frac{X*Y}{ X Y} = \frac{{\sum x_{n} y_{n} }}{{\sqrt {\sum x_{n}^{2} } \times \sqrt {\sum y_{n}^{2} } }}$$

#### Patient similarity

The weighted sum of the four feature similarities was used as the single measure of patient similarity (PS) for patients *i* and *j*:

9 $${\text{PS}}\left( {i,j} \right) = w_{1} *\left[ {{\text{FS}}_{{{\text{D}}1}} \left( {i,j} \right) \;{\text{or}}\; {\text{FS}}_{{{\text{D}}2}} \left( {i,j} \right)} \right] + w_{2} *{\text{FS}}_{L} \left( {i,j} \right) + w_{3} *{\text{FS}}_{A} \left( {i,j} \right) + w_{4} *{\text{FS}}_{S} \left( {i,j} \right),$$

where 0 ≤ *w*_1_–*w*_4_ ≤ 1 (Σ*w*_*i*_ = 1) are the weights of the four feature similarities. In the current study, *w*_1_–*w*_4_ were assigned to 0.4, 0.4, 0.1, and 0.1, respectively, which were determined experimentally in our previous study [29].

### Application of patient similarity

#### Data source

EMR data used in this study were derived from all inpatients discharged from a tertiary hospital in Beijing, China between 2014 and 2016. Individual hospitalizations were de-identified and maintained as unique records, including age at admission, sex, disease diagnoses at discharged (up to 11), and laboratory tests during hospitalization. Disease diagnoses were identified using ICD-10 codes.

Records for patients who had disease diagnoses with ICD-10 codes starting with O (complications of pregnancy), P (certain conditions originating in the perinatal period), S and T (incidental conditions such as poisoning and injuries), and Y and V (supplementary classification codes) were excluded. In addition, for patients with more than one hospitalization (i.e., readmission), records for follow-up admissions were excluded to maintain a study dataset containing distinct patients.

In one hospitalization episode, patients are not necessary to take all laboratory tests, leading to a large number of missing values in laboratory test fields. This will make it more difficult to compute feature similarity for laboratory test. Therefore, records with more missing laboratory tests should be excluded in the current study. For the task of disease prediction, DM (ICD-10 codes of E10–E14 [30, 31]) was chosen as the target disease. Thus, 77 most regular laboratory test items related to DM, including blood test, urine test and electrolyte test were employed for the similarity computation. Records with missing values of any of the above 77 laboratory test items were then excluded.

In total, 8245 patients with any diabetes diagnosis (positive samples) remained and another 8245 patients without any diabetes diagnoses (negative samples) were randomly selected, giving a study dataset of 16,490 samples (Additional file 1: Figure S2). The mean ages of the patients with and without DM were 63.0 ± 11.6 years and 57.2 ± 17.1 years (*t*-test, *P* < 0.001), respectively. 5163 (62.6%) patients in DM group were males, whereas 6062 (73.5%) in non-DM group (*χ*^2^ test, *P* < 0.001).

#### Machine learning models

For an index (test) patient with an unknown label, a personalized predictive model was built based on the most similar patients from the training samples. This model was then tested on the index patient. This study predicted the index patient as diabetic or not diabetic, which was a binary classification problem. To explore the impact of the model on the performance of the similarity-based predictive model, three machine learning-based classification models with disparate algorithms and structures were used: *k*NN, LR, and RF classifiers.

In our classification setting, the *k*NN classifier assigned each index patient with the majority class of its *k* (*k *= 50 in this study) nearest labeled neighbors, based on Euclidean distance from the training set [32]. The probability of that patient being predicted as diabetic was defined as the proportion of patients with diabetes among the *k* neighbors. LR is a discriminative model in machine learning, or a kind of generalized linear model with a logit link function and binomial distribution [32]. The predicted outcome of the LR classifier for the index patient was the probability of belonging to the positive class. RF [33] is an ensemble classifier consisting of many decision trees (100 trees in this study) based on random feature selection [34, 35] and bootstrap aggregation [36]. The final predicted probability of belonging to each class for the index patient was obtained by combining the predictions of individual trees.

Input features for the classification models were age, sex, disease diagnoses and 77 laboratory tests. To reduce the dimensionality of the feature space, diseases that occurred in less than 1% of the study dataset were ruled out. In total, 27 diseases with a statistically different occurrence rate between patients with and without DM (*χ*^2^ test, *P* < 0.05) remained for further modeling. Finally, 106 features were used as the input features for the models.

#### Performance evaluation

We used a hold-out method to validate the predictive models. All the 8245 patients with DM were split randomly into a set of 5000 samples and a set of 3245 samples for training and test, respectively. Accordingly, 5000 and 3245 patients without DM were selected randomly to be used as training and test samples, respectively. As a result, the final study population was consisted of 16,490 samples, 10,000 of them were used as the training samples and the rest 6490 samples as the test samples. The basic characteristics of samples both in the training set and test set were presented in Table 1. The characteristics included age, sex, several major chronic diseases according to the Charlson comorbidities [37] and expert’s advice (such as heart disease, pulmonary disease, liver disease, and hypertension), and two laboratory test items (i.e., serum glucose and urine glucose) related to diabetes diagnosis. There were no statistical differences between the two groups in these characteristics.

**Table 1:** The basic characteristics of samples in the test set and training set

Characteristic	Test set (*n* = 6490)	Training set (*n* = 10,000)	*P* value^#^
Male gender, *n* (%)	4387 (67.6%)	6838 (68.4%)	0.282
Age (years), mean ± SD	60.1 ± 14.7	60.1 ± 15.0	0.967
Myocardial infarction, *n* (%)	443 (6.8%)	656 (6.6%)	0.615
Congestive heart failure, *n* (%)	507 (7.8%)	795 (8.0%)	0.642
Chronic obstructive pulmonary disease, *n* (%)	288 (4.4%)	467 (4.7%)	0.368
Mild liver disease, *n* (%)	799 (12.3%)	1301 (13.0%)	0.188
Hypertension, *n* (%)	3501 (53.9%)	5389 (53.9%)	0.950
Coronary heart disease, *n* (%)	2206 (34.0%)	3331 (33.3%)	0.366
Serum glucose (mmol/L), mean ± SD	6.6 ± 2.9	6.7 ± 2.9	0.793
Abnormal urine glucose, *n* (%)	1222 (18.8%)	1884 (18.8%)	0.987

^#^Pearson’s *χ*^2^ test for nominal variables and *T*-test for scale variables

*SD* standard deviation

To dynamically evaluate the potentials of the proposed patient similarity when being used in selecting similar samples for predicting diabetes, predictive models were trained based on top *K* similar patients, where the smaller the sample size *K*, the more similar the selected training patients. Performance evaluation and comparisons were then conducted among the three classification models built on similar and randomly selected samples with the same sample size, and the changing trends of the predictive performance as the size of the training samples increased could be analyzed. Predictive performance was evaluated by the AUC. The cubic polynomial fitting was used to give the changing trends of AUCs.

To help understand the classification process of the *k*NN model, the patient to be predicted and its *k* (*k* = 10, 50, 100, respectively) nearest neighbors were visualized. Another visualization was used to show the top 20 important features captured by the RF models which were built on similar patients and randomly selected patients, separately. Feature importance was determined by the Gini coefficients.

All computations and analyses were conducted using R 3.4.0 software (https://cran.r-project.org/).

## Supplementary information

**Additional file 1: Figure S1.** Partial view of the hierarchy system of the International Classification of Diseases, tenth revision. **Figure S2.** A flow chart of the record selection. DM, diabetes mellitus.

## Data Availability

Not applicable.

## Abbreviations

AUC: area under the ROC curve
CCS: Clinical Classification Software
DM: diabetes mellitus
EMR: electronic medical records
ICD-10: International Classification of Diseases, tenth revision
*K*NN: *k*-nearest neighbor
LR: logistic regression
NCA: nearest common ancestor
SD: standard deviation
RF: random forest
ROC: receiver operating characteristic
