# Supplementary material for: Measurement and application of patient similarity in personalized predictive modeling based on electronic medical records
Source: Biomed Eng Online. 2019 Oct 11;18:98. doi: 10.1186/s12938-019-0718-2 (PMC6788002; doi:10.1186/s12938-019-0718-2)
Supplement: Supplementary file 1 — Additional file 1: Figure S1. Partial view of the hierarchy system of the International Classification of Diseases, tenth revision. Figure S2. A flow chart of the record selection. DM, diabetes mellitus. [file 12938_2019_718_MOESM1_ESM.pdf]

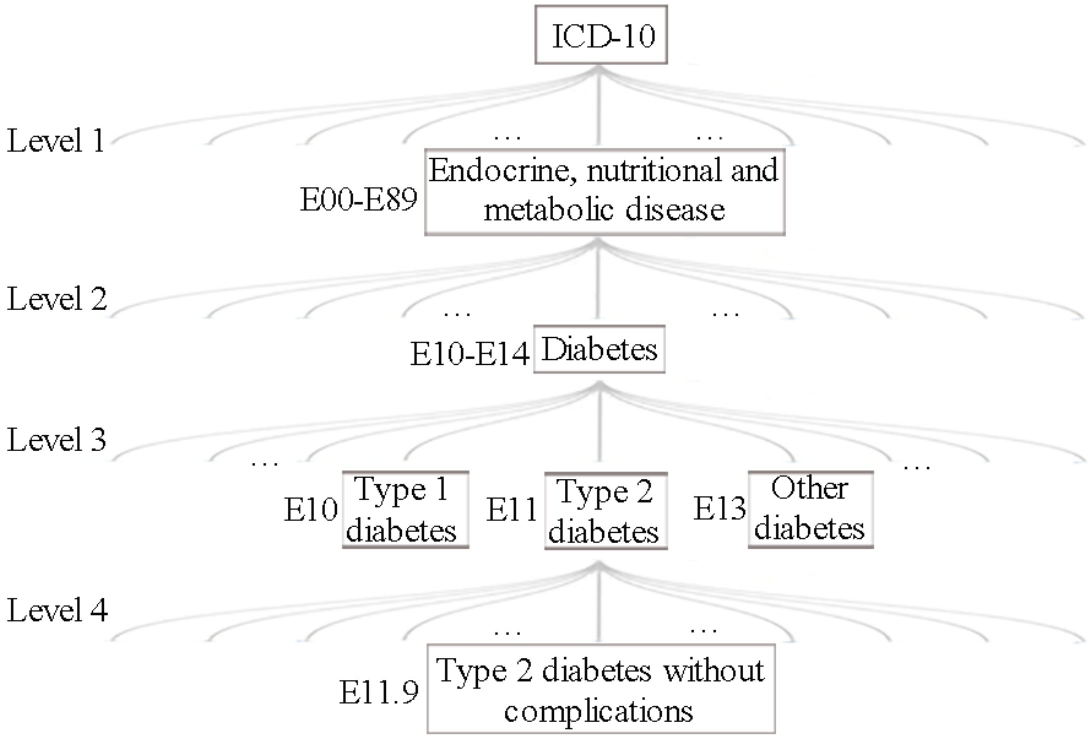

Fig. S1. Partial view of the hierarchy system of the International Classification of Diseases, tenth revision.

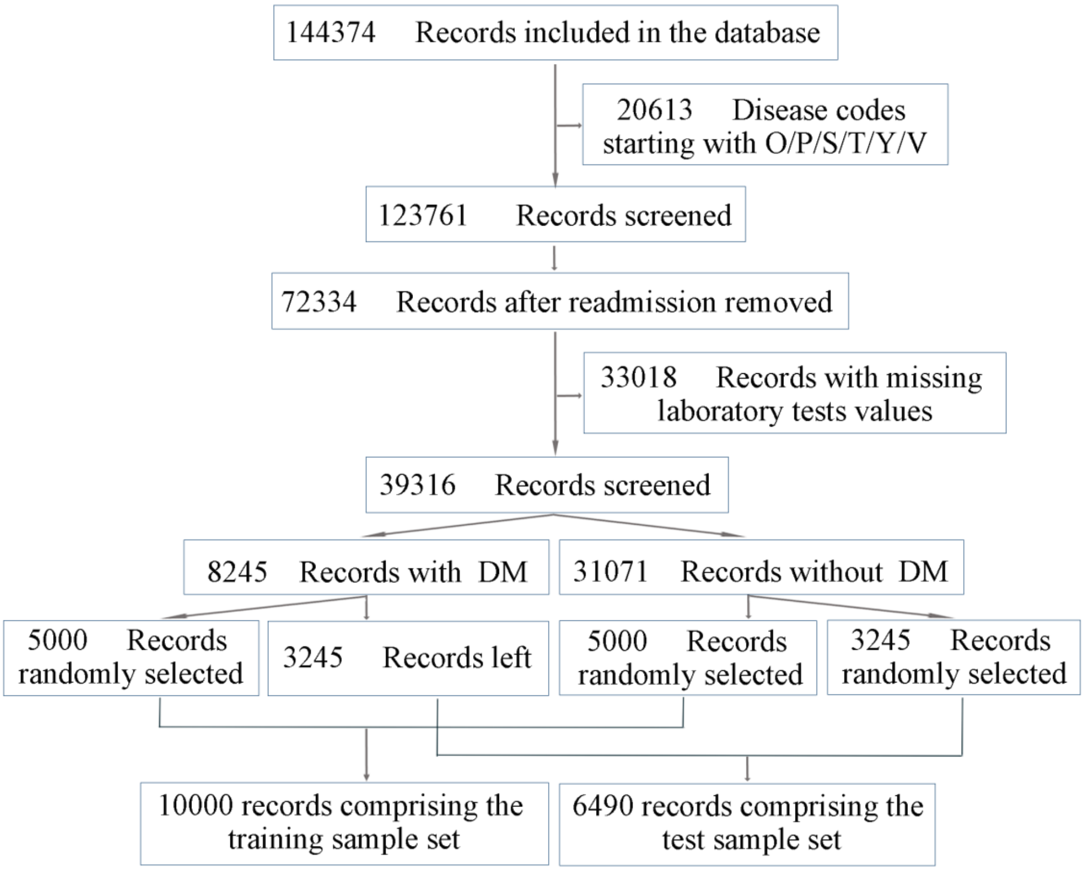

Fig. S2. A flow chart of the record selection. DM, diabetes mellitus.
